# The influence of cell culture media and their additives on virus inactivation in vitro

**DOI:** 10.3205/dgkh000602

**Published:** 2025-11-28

**Authors:** Florian H. H. Brill, Britta Becker, Dajana Paulmann, Birte Bischoff, Anke Herrmann, Toni Luise Meister, Eike Steinmann, Jochen Steinmann, Katharina Konrat

**Affiliations:** 1 Dr. Brill + Partner GmbH, Institute for Hygiene and Microbiology, Bremen, Germany; 2 Robert Koch-Institute, Hospital Hygiene, Infection Prevention and Control, Berlin, Germany; 3 Department for Molecular & Medical Virology, Ruhr-University Bochum, Germany; 4 Institute for Infection Research and Vaccine Development, University Medical Center Hamburg-Eppendorf, Hamburg, Germany; 5 Department for Clinical Immunology of Infectious Diseases, Bernhard Nocht Institute for Tropical Medicine (BNITM), Hamburg, Germany; 6 German Center for Infection Research (DZIF), Partner site Hamburg-Lübeck-Borstel-Riems, Hamburg, Germany

**Keywords:** murine norovirus, EN 14476, virucidal activity, cell culture media, additives

## Abstract

**Aim:**

Comparative inactivation studies with the murine norovirus (MNV) and different test substances showed considerable different results between test laboratories. To decipher the underlying mechanisms of this observation, different virus pools (virus suspensions) of the MNV were analysed in two independent laboratories by performing inactivation tests with i. a. various alcoholic formulations and different culture media.

**Methods:**

Virucidal activity of different test formulations (propan-2-ol, propan-1-ol, ethanol, aldehydes and peracetic acid) against MNV has been tested in the quantitative suspension test according to EN 14476:2019.

**Results:**

Results of the inactivation studies with MNV varied greatly depending on the cell culture medium and its additives used. In particular, the addition of HEPES to the medium in the course of virus propagation led to a strong decrease in the virus inactivation, when this virus pool was used in the approach with an alcoholic formulation.

**Conclusion:**

The use of different cell culture media with individual supplements can have a major impact on the results of inactivation studies with MNV. Therefore, harmonized protocols of viral inactivation test should be developed that describe the same cell culture conditions.

## Zusammenfassung

**Zielsetzung:**

In früheren vergleichenden Inaktivierungsstudien mit dem murinen Norovirus (MNV) und verschiedenen Testsubstanzen ergaben sich erhebliche Unterschiede in den Ergebnissen zwischen verschiedenen Prüflaboratorien. Um dafür die Ursache zu finden, wurden verschiedene Viruspools (Virussuspensionen) des MNV in zwei unabhängigen Laboratorien analysiert. Hierzu wurden Inaktivierungstests u.a. mit verschiedenen alkoholischen Formulierungen und unterschiedlichen Zellkulturmedien durchgeführt.

**Methoden:**

Die viruzide Wirksamkeit verschiedener Testformulierungen (Propan-2-ol, Propan-1-ol, Ethanol, Aldehyde und Peressigsäure) gegen das MNV wurde im quantitativen Suspensionstest gemäß EN 14476:2019 geprüft.

**Ergebnisse:**

Die Ergebnisse der Inaktivierungsstudien mit dem MNV variierten stark in Abhängigkeit vom verwendeten Zellkulturmedium und dessen Zusätzen. Insbesondere die Zugabe von HEPES zum Medium im Verlauf der Virusvermehrung führte zu einer starken Abnahme der Virusinaktivierung, wenn dieser Viruspool im Ansatz mit einer alkoholischen Formulierung verwendet wurde.

**Schlussfolgerung:**

Die Verwendung von unterschiedlichen Medien mit individuellen Zusätzen können einen großen Einfluss auf die Ergebnisse von Inaktivierungsstudien mit dem MNV haben. Daher sollten harmonisierte Protokolle für Virusinaktivierungstests entwickelt werden, die dieselben Zellkulturbedingungen beschreiben.

## Introduction

Chemical disinfectants with proven virus inactivating properties are used in hospitals and other medical settings to interrupt infection chains. These disinfectants should be able to disinfect depending form the formulation e.g. hands, surfaces, instruments, and laundry. Their virucidal activity (reduction of virus titres by at least 4 log steps) is tested according to national and international guidelines with different test viruses with and without envelope in a quantitative suspension test, such as the guideline of DVV/RKI [1] or the EN 14476 standard [2], followed by tests under practical conditions, like EN 16777 [3] and EN 17111 [4]. 

In general, the different guidelines standardize many test parameters such as test viruses, test temperature, exposure time, soil load, and the internal validation controls. They also provide a detailed description of the testing procedure. At the same time, however, the standards allow a certain amount of flexibility e.g. in the production of the test virus suspension, the cell lines used for virus propagation and endpoint titration, and finally the respective cell culture media and their supplements used for the different systems. 

This raises the question of whether this flexibility could cause and/or explain different results in inactivation studies between different testing laboratories, when using the same test viruses and the same test substances.

In this study, two independent laboratories (Lab 1 and Lab 2) compared the influence of different cell culture media, used for virus propagation and cell culture, on the results of suspension tests according to EN 14476:2019 with the murine norovirus (MNV) as non-enveloped test virus and different test substances. The results obtained showed large differences in virus reduction depending on the cell culture medium used and, in particular, its supplements.

## Materials and methods

### Cells and culture media

All experiments were performed in two laboratories located in Germany (Lab 1 and Lab 2) with RAW 264.7 cells, obtained from the American Type Culture Collection (ATCC TIB-71). For cell cultivation and the suspension tests, the following cell culture media were used: a) Dulbecco’s Modified Eagle Medium High Glucose (4.5 g/l) (DMEM) (biowest, art.-no. L0106-500) and b) a mixture of Eagle’s minimum essential medium with Earle’s Balanced Salt (EMEM) (gibco, art.-no. 61100) and Eagle’s minimum essential medium (MEM) (Sigma, art.-no. M4642) => EMEM/MEM (1:1). 

The respective culture media were used with different additives. DMEM was supplemented with L-glutamine (biowest, art.-no. X0550), non-essential amino acids (NEA) (Sigma, art.-no. M7145), sodium pyruvate (Sigma, art.-no. S8636), and fetal calf serum (FCS) from biowest (art.-no. S1600) (10% for subcultivation and 5% FCS in a final concentration per well for suspension tests). Thereby, DMEM was used with and without 2-(4-(2-Hydroxyethyl)-1-piperazinyl)-ethansulfonacid (HEPES) (Sigma, art.-no. H0887). EMEM/MEM (1:1) was supplemented with sodium bicarbonate 2.2 g/L (Merck/Supelco, art.-no. 1.06329.1000), non-essential amino acids (NEA) (PAN Biotech, art.-no. P08-32100), sodium pyruvate (PAN Biotech, art.-no. P04-43100), and fetal calf serum (FCS) from (biowest, art.-no. S181G-500) (10% for subcultivation and 2% FCS in a final concentration per well for suspension tests). 

### Virus propagation

Virus suspensions were prepared by replicating the murine norovirus (MNV), strain S99 (RVB 0651, obtained from Friedrich-Loeffler-Institute, Greifswald, Insel Riems, Germany) in RAW 264.7 cells. The two laboratories used both DMEM as well as EMEM/MEM (1:1) with different supplements, depending on the test performed, as indicated in the results. A subconfluent monolayer of RAW cells was inoculated with MNV and incubated at 37°C in 5% CO_2_. After the cytopathic effect (CPE) became evident, cells were lysed by freeze-thaw cycle and virus suspension (virus pool) was harvested by removing cell debris by low speed centrifugation and stored in aliquots at –80°C. 

### Test formulations

The formulations to be tested against MNV were prepared from commercially available products using Aqua dest., PBS (formaldehyde) or standardized hard water (propan-2-ol; Lab 2 only) as diluent at various concentrations (see Table 1 (Tab. 1)). 

### Determination of virucidal activity

In both laboratories tests were performed according to EN 14476:2019 [2] under clean conditions at 20°C. In general, 1 part of the prepared virus suspension was mixed with 1 part of 3 g/L bovine albumin (BSA) and 8 parts of the respective test formulation or 8 parts of aqua bidest (or hard water for propan-2-ol (Lab 2)) for the virus control (exception: 0.7% formaldehyde was prepared as described in the EN 14476). In some experiments, the virus suspension was mixed with varying amounts of HEPES or 3[N-morpholino]propane sulfonic acid (MOPS) buffer (ThermoFisher, art.-no. J61821) before preparing the actual inactivation mix.

After the respective contact time, infectivity was stopped by immediate serial dilutions (1:10 dilutions) with ice-cold medium. The virus titres were calculated by applying 100 µL of each dilution in eight (Lab 1) or ten (Lab 2) wells of a 96-well microtitre plate containing 100 µL of cell suspension (endpoint dilution assay), followed by an incubation of the microtitre plate at 37°C in 5% CO_2_ until a cytopathic effect could be detected.

The respective virus titres were determined using the method of Spearman [5] and Kaerber [6] and expressed as lg TCID_50_/mL. The reduction factors (RF) were calculated as the difference between the virus titre after exposure to the formulation analyzed and the titre of the corresponding virus control (assay performed as described above but with water instead of test product). 

## Results

### Influence of different culture media for virus propagation on the virucidal activity of 70% propan-2-ol

In the past, different results were reported from MNV inactivation studies according to EN 14476 (especially with alcoholic test substances). To find the reason, the culture media and all corresponding additives (including FCS) routinely used for this system were exchanged between the two laboratories involved in this study. Lab 1 normally used DMEM with 4.5 g/L glucose and its additives L-glutamine, NEA, sodium pyruvate, and HEPES and FCS for cell culture. Lab 2 commonly used EMEM/MEM with L-glutamine (1:1), NEA and sodium pyruvate as supplements and FCS.

**Figure 1:**
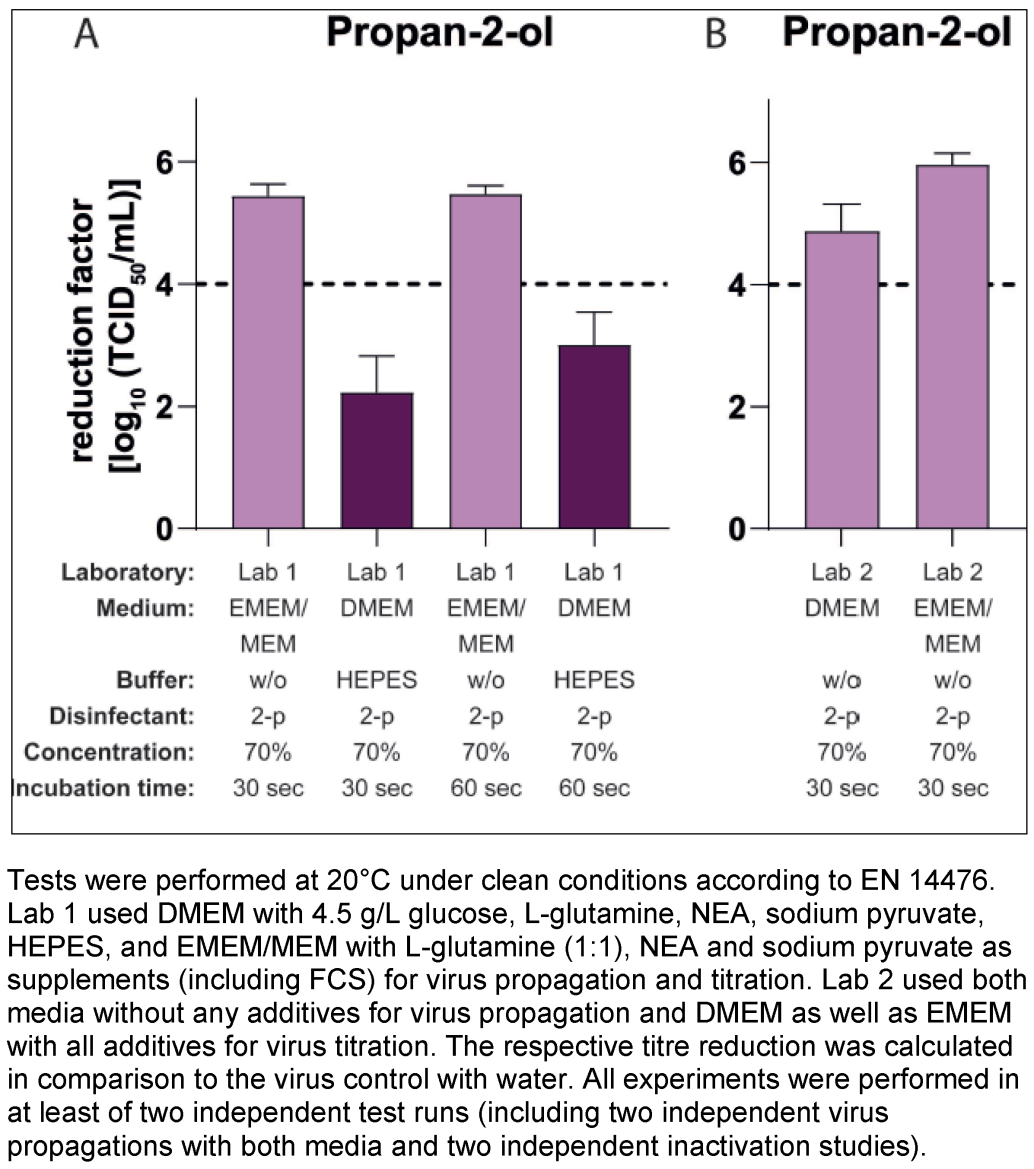
Influence of different cell culture media on the inactivation of murine norovirus (MNV) by 70% propan-2-ol

Both laboratories produced new virus pools under their own conditions and with their own RAW cells and stock virus and their own medium in parallel with the other laboratory’s medium. For the first experiments, Lab 1 used the DMEM and the EMEM/MEM with all additives as described above and 2% of the respective FCS for virus propagation. Lab 2 prepared the MNV suspension without any supplements in both media. The virus pools produced were then used for an inactivation study in the suspension test with 70% propan-2-ol as the final concentration. The results are shown in Figure 1 (Fig. 1). 

Lab 1 achieved a reduction factor (RF) of 2.22 lg after 30 seconds exposure using DMEM (with all described additives including FCS) as the culture medium for virus propagation and inactivation tests. In contrast, with the EMEM/MEM medium (with the respective additives received from Lab 2) for virus propagation and titration, a RF of 5.44 lg after 30 s of exposure could be demonstrated (Figure 1A (Fig. 1)).

In contrast, in Lab 2 only low differences in the reduction factors were determined, when using DMEM or EMEM/MEM without additives for virus propagation (Figure 1B (Fig. 1)). In summary, these results show that obviously specific additives are responsible for the differences obtained.

### Influence of FCS and HEPES in cell culture media for virus propagation on the virucidal activity of 70% propan-2-ol

Next, the effect of the FCS as an important and essential supplement in media during cell culture procedures was investigated as a possible cause. Therefore, pre-cultivation of the RAW cells, virus propagation, suspension test and titration were performed with both media as described above, but instead of the respective FCS for each medium, the same FCS was used for both media (Lab 1) or the medium with and without FCS (Lab 2) during virus pool production. Both laboratories achieved almost the same results as described above in the inactivation studies with 70% propan-2-ol with these new virus pools (data not shown), indicating, that FCS had no effect on the results. 

**Figure 2:**
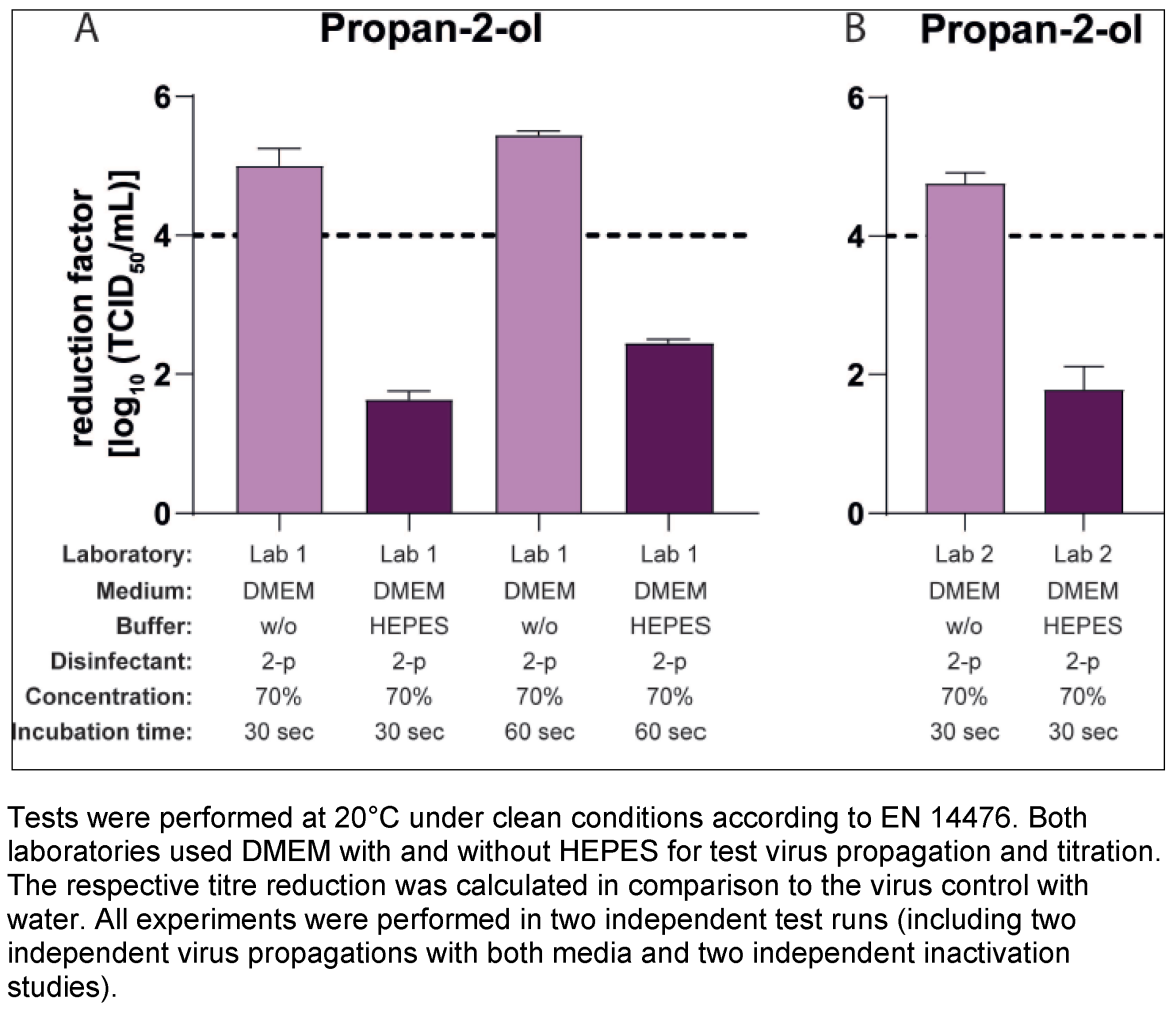
Influence of HEPES buffer in the medium during virus propagation on the inactivation of murine norovirus (MNV) by 70% propan-2-ol

In the next step, the impact of HEPES, a commonly used buffer for cell culture medium was analysed. Suspension tests and titration of the respective dilutions were performed as in the previous studies, but virus production was carried out in both laboratories using only DMEM as the cell culture medium with or without HEPES. As shown in Figure 2 (Fig. 2), the addition or removal of the buffer during the virus propagation had a major impact on the results of the inactivation tests with propan-2-ol against MNV. The mean RFs for the systems using the medium with HEPES were 1.63 lg (Lab 1, Figure 2A (Fig. 2)) and 1.78 lg (Lab 2; Figure 2B (Fig. 2)) after 30 s of incubation and the mean RF for the systems without HEPES were 5.00 lg (Lab 1) and 4.76 lg (Lab 2) (Figure 2A (Fig. 2) and Figure 2B (Fig. 2)).

### Influence of HEPES in the culture media for virus propagation on the virucidal activity of additional active substances

**Figure 3:**
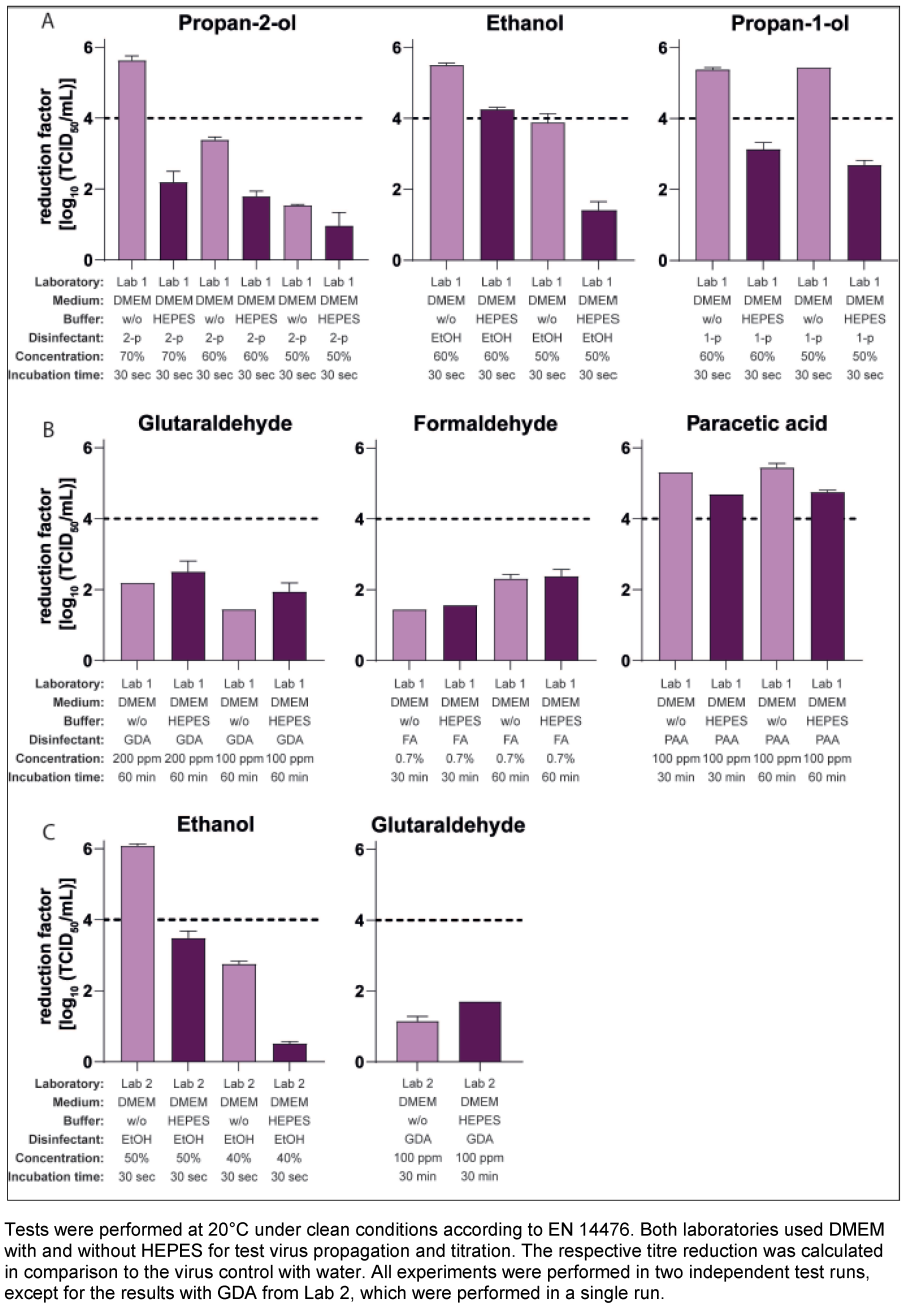
Influence of HEPES buffer in the medium during virus propagation on the inactivation of murine norovirus (MNV) by different active substances

To investigate whether HEPES could also affect the results of other active ingredients commonly used in disinfectants, further inactivation studies were performed according to EN 14476 with the same virus pools (generated in media with and without HEPES) and varied active formulations and concentrations. Beside the 70% v/v propan-2-ol solution tested so far, also significant differences in the results with a 60% v/v propan-2-ol solution as well as with other alcohols, as 50% v/v ethanol and 50% and 60% v/v propan-1-ol, could be shown in Lab 1 (Figure 3A (Fig. 3)). In addition, a slight effect with 100 ppm PAA as test substance could be detected, whereas no differences in the results could be shown testing formaldehyde or GDA (Figure 3B (Fig. 3)). Lab 2 produced similar results with significant differences in the reduction factors with 40% and 50% w/w ethanol as the test concentration, with no significant differences measured with GDA (Figure 3C (Fig. 3)) and 30% w/w ethanol (data not shown).

### Impact of the addition of HEPES or MOPS to the virus suspension

**Figure 4:**
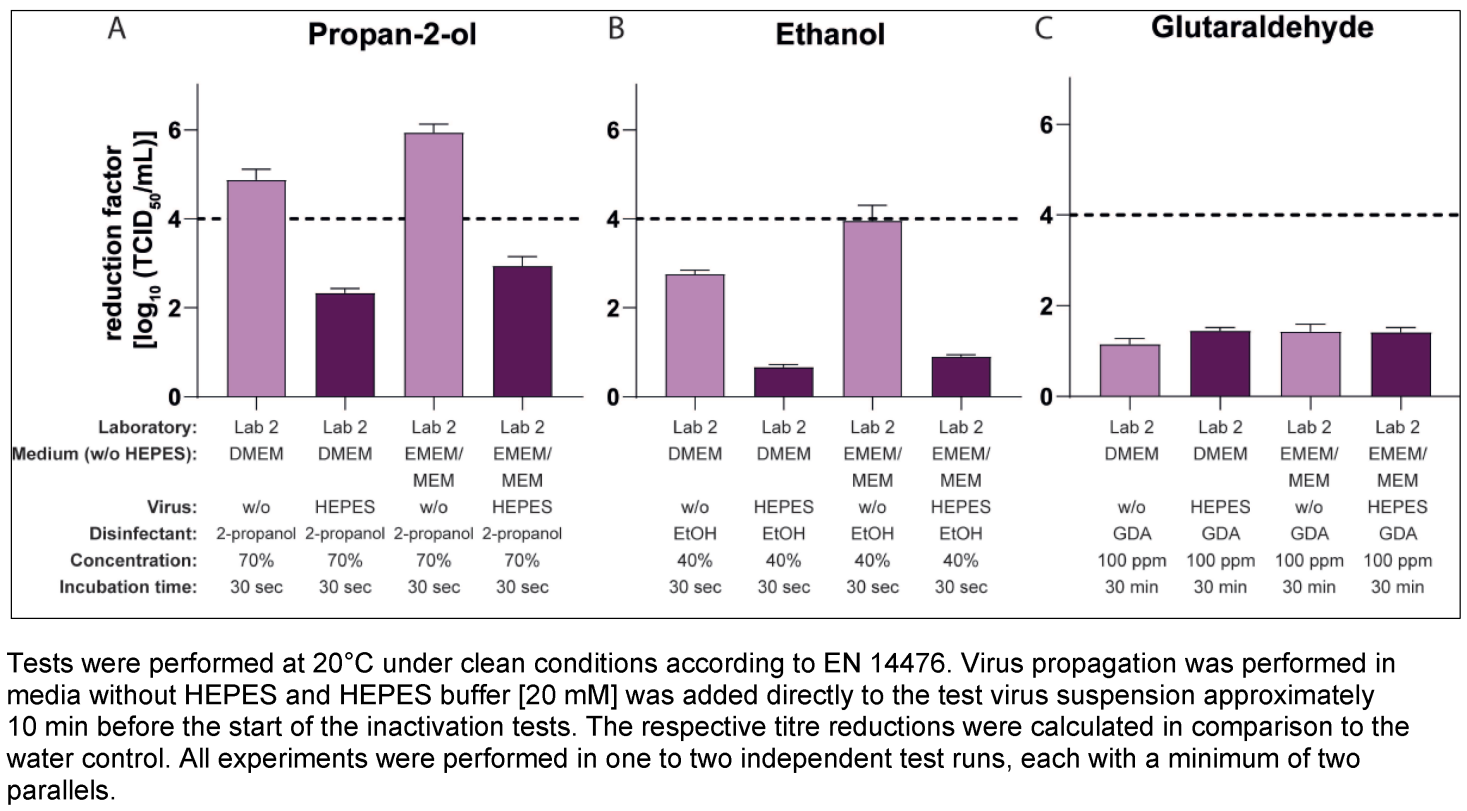
Influence of HEPES buffer after pre-incubation with the virus suspension on the inactivation of murine norovirus (MNV) by different active substances

The final experiments tested whether adding a buffer directly to the virus suspension before an inactivation test had the same effect on the results as adding the buffer in the media during virus propagation. Lab 2 performed these tests with 70% v/v propan-2-ol, 40% ethanol w/w and 100 ppm GDA with virus pools propagated in DMEM or EMEM/MEM without buffer (and without FCS), whereby HEPES at a concentration of 20 mM was added to the virus suspension approximately 10 min before the inactivation test started. The results are presented in Figure 4A (Fig. 4), Figure 4B (Fig. 4), and Figure 4C (Fig. 4). Testing 70% v/v propan-2-ol, the mean reduction factor using the virus suspension without HEPES (EMEM/MEM: RF 5.94 lg; DMEM: RF 4.88 lg) decreased to 2.94 lg (EMEM/MEM) and 2.33 lg (DMEM) after the addition of 20 mM HEPES to the virus suspension (Figure 4A (Fig. 4)). Similar results could be obtained with 40% w/w ethanol (Figure 4B (Fig. 4)) and no effect on the results with GDA could be observed (Figure 4C (Fig. 4)). 

**Figure 5:**
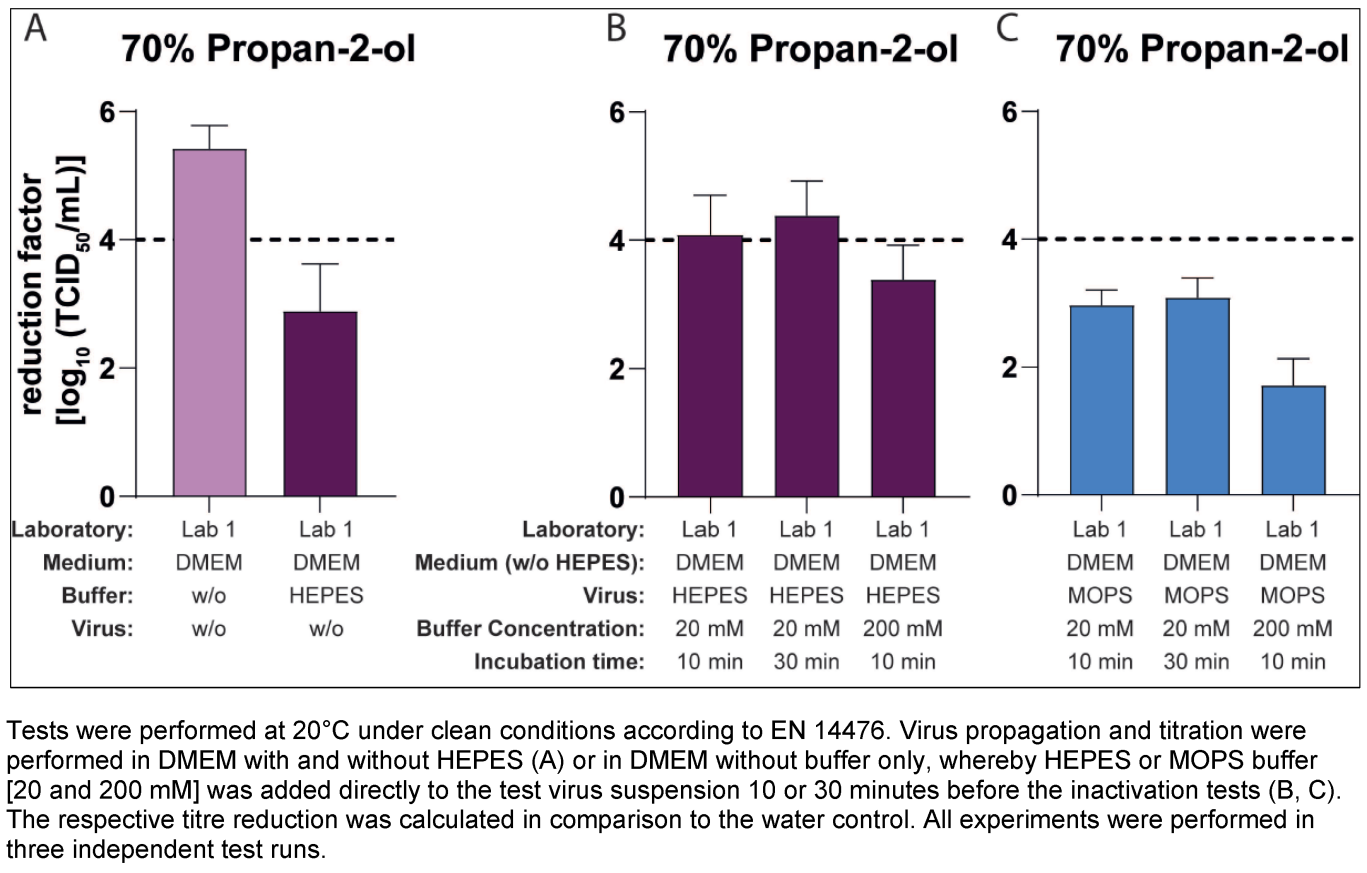
Influence of HEPES or MOPS buffer after pre-incubation with the virus suspension on the inactivation of murine norovirus (MNV) by 70% propan-2-ol

Lab 1 performed these tests using only 70% v/v propan-2-ol and a virus pool propagated in DMEM without buffer (with FCS). HEPES and, in an additional approach, MOPS, which is also commonly used as a buffering agent in cell culture, were added to the test virus suspension at concentrations of 20 and 200 mM for 10 and 30 minutes before the inactivation assay. As a control, approaches were performed with the “pure” virus suspensions propagated in DMEM with or without HEPES. The mean reduction factor of the control (virus pool production without HEPES and the pure virus suspension) was 5.42 lg after 30 s of exposure (Figure 5A (Fig. 5)). In contrast, after 10 min of pre-incubation of the same virus suspension with HEPES this RF decreased to 4.08 lg (addition of 20 mM HEPES) and 3.38 lg (addition of 200 mM HEPES) (Figure 5B (Fig. 5)). Even after the addition of MOPS buffer, there was a strong decrease in the RF to 2.96 lg (20 mM) and 1.71 lg (200 mM). Prolonging the pre-incubation to 30 min with 20 mM buffer had no significant effect (Figure 5B (Fig. 5) and Figure 5C (Fig. 5)). 

However, not only the presence of HEPES during pool preparation itself, but also the subsequent addition of HEPES or MOPS to a pure virus suspension (propagated in medium without HEPES) can have a strong impact on the results of inactivation studies.

## Discussion

The various basic media routinely used for the cultivation of cell lines contain nutrients intended to ensure growth of the cells in culture. The ready-to-use composition of these media with their specific ingredients and individual additives (including FCS) is often cell type-specific and is also determined, among other things, by the type and manner of culture conditions (e.g. duration of cultivation or cultivation with and without CO_2_ supply). 

As the current standards for performing virucidal tests do not yet contain any regulations on the medium used or the cells to be used for a specific test virus, the different systems consisting of test virus and cell line are generally established by the various test laboratories themselves on the basis of successful virus propagation to obtain high titres. This means that, when testing disinfectants for viral activity, different media and additives as well as different cells could be used with MNV as test virus in the different test laboratories. In this study, it could be shown for the first time that the choice and composition of cell culture media can have a significant influence on the results of virucidal tests with MNV according to EN 14476 and HEPES was identified as the decisive factor. 

HEPES is actually classified as a zwitterionic “GOOD” buffer and is routinely added to the medium at concentrations ranging from 10 to 25 mM if, for example, the buffer capacity of the bicarbonate in a medium is no longer sufficient (e.g. when cell cultures are cultivated without CO_2_ in a closed system or stored outside the CO_2_ incubator for a longer period of time) [7], [8].

However, if HEPES was contained in the cell culture medium (in this case DMEM) during virus propagation, this led to a drastic drop in the reduction factors after just 30 seconds in the inactivation assays with 70% v/v propan-2-ol. The difference in the mean RF without and with buffer in the medium amounted to Δ3.22 (Figure 1A (Fig. 1)) and Δ3.37 (Figure 2A (Fig. 2)) or Δ2.98 lg levels (Figure 2B (Fig. 2)). Such significant differences were not limited to results with propan-2-ol, but could also be obtained with other alcoholic formulations in the MNV inactivation assays, such as ethanol or propan-1-ol (Figure 3A (Fig. 3) and Figure 3C (Fig. 3)). Of note, some of the test concentrations of the alcoholic formulations were too low or too high so that the differences were difficult or impossible to visualize because of the excess or lack of activity. This may also apply to the inactivation studies with 100 ppm PAA against the MNV. Here, the RF was above 4 lg steps for all approaches and only slight differences could be detected when using virus suspensions from a culture with and without HEPES (Figure 3B (Fig. 3)). To detect a possible effect, further studies with PAA at a lower concentration (e.g. 50 ppm) would be necessary. In contrast, in the studies with GDA and formaldehyde, there were no visible effects (regardless of whether HEPES was added to the DMEM during virus cultivation or not, the reduction factors achieved were almost identical (Figure 3B (Fig. 3)).

In the past, a number of findings have already been made in connection with the use of HEPES in cell culture media, which describe further advantages and/or disadvantages in addition to its efficient buffering capacity. For instance, the presence of HEPES in cell culture medium promotes the uptake and transfection of proteins [9], [10]. The buffer can also be taken up by cells through endocytosis and thus influence various intracellular processes [11], [12], [13]. Hugel et al. [14] also showed the pH-dependent inhibition of GABAA receptors by HEPES, presumably through protonation of the buffer. This means that it is quite conceivable that the buffer substance could react directly with an individual active substance and thus inhibit it. It is also possible that HEPES interacts directly with the virus and that the virus is thus better protected against the attack by an active substance. The final inactivation studies carried out with HEPES and MOPS as a second buffer substance, in which only the virus (from propagation without buffer) was spiked with HEPES or MOPS [20 mM or 200 mM] shortly before the start of the experiment, do not provide a definitive explanation for the effects described. 

However, results show that significant differences in the reduction factors can occur not only after virus propagation in medium with and without buffer, but also after propagation of a MNV pool without HEPES and the subsequent addition of the buffer to the virus. In Lab 2, the RF after an exposure time of 30 seconds in the preparations without HEPES were 2.73 lg to 3.00 lg (70% v/v propan-2-ol) and 2.08 lg to 3.05 lg (40% w/w ethanol) lg levels higher than in the preparations in which the virus was mixed with the HEPES buffer (Figure 4 (Fig. 4)). This means that the difference between the preparations with and without HEPES was close to the results achieved so far in the laboratory. In Lab 1, the results with 20 mM HEPES in the virus buffer mixture and 70% v/v propan-2-ol were not quite as clear, even if the effect was further enhanced by increasing the buffer concentration to 200 mM (Figure 5(Fig. 5)). However, it was interesting to note that after the addition of the MOPS buffer to the virus suspension, even higher effects were detected than with the use of the HEPES virus mixture (Figure 5C (Fig. 5)). 

If the buffer substance would be attached directly to the virus itself, then the direct addition of HEPES to the virus in both laboratories should have given roughly similar results, comparable to the results of the previous experiments with and without HEPES during virus propagation. However, this was not the case in Lab 1.

Thus, the cause(s) or mechanism(s) ultimately responsible for the results of the MNV inactivation studies with or without HEPES (or MOPS) remain still unclear. It is possible, that the reported effect may be a combination of an interaction between the virus and the buffer, in which the virus replication itself also plays a role, and an interaction of the buffer with the test substance. But this should be the subject of further investigations.

In our study, HEPES is the first cell culture additive to be identified that can significantly influence the results of inactivation studies with MNV, and thus the resulting activity of disinfectants, depending on the test substance and its concentration. At this time, it is not possible to assess which viruses this ultimately concerns or which specific factors in other systems may influence the results of virucidal tests individually. But the data obtained impressively illustrate the importance of better standardisation of the test specifications in guidelines and norms to ensure that results are comparable even between different test laboratories. Thereby, it is not only the standardisation of the used cell culture media and their additives that is crucial for valid virucidal inactivation tests, but it may also be necessary to determine the cell line for the respective replication system. 

## Notes

### Authors’ ORCIDs

- Brill FHH: https://orcid.org/0000-0001-9681-8752
- Meister TL: https://orcid.org/0000-0001-8962-9443
- Steinmann E: https://orcid.org/0000-0002-3654- 9965
- Steinmann J: https://orcid.org/0000-0002-0527-8840
- Konrat K: https://orcid.org/0000-0001-9963-5222

### Acknowledgments

The authors would like to thank the members of the Commission for Virus Disinfection of the German Association for the Control of Virus Diseases (DVV) e.V. and the German Society of Virology e.V., in particular Ingeborg Schwebke, Sven Reiche and Maren Eggers, for their contributions to the earlier comparative inactivation studies, and Sandra Niendorf and Dirk Höper for the corresponding genome sequencing and analysis (data not shown).

### Competing interests

JS works as a consultant for Dr. Brill + Partner GmbH Institute for Hygiene and Microbiology. The other authors declare that they have no competing interests.

## Figures and Tables

**Table 1:**
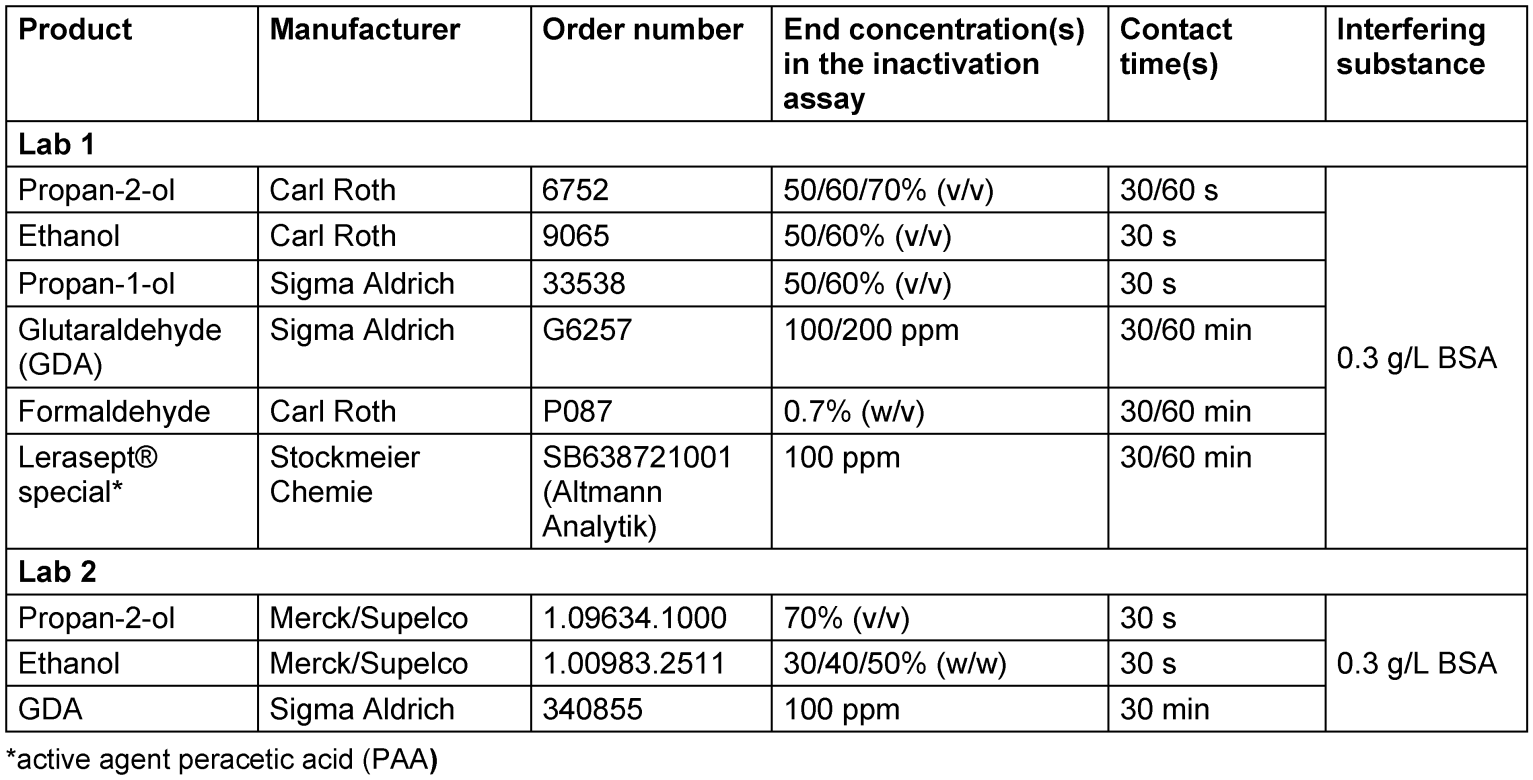
Overview of the test products and conditions used in the study
